# Optimized Adsorption–Catalytic Conversion for Lithium Polysulfides by Constructing Bimetallic Compounds for Lithium–Sulfur Batteries

**DOI:** 10.3390/ma17133075

**Published:** 2024-06-22

**Authors:** Liping Chen, Runhua Wang, Nan Li, Yang Bai, Yimo Zhou, Juan Wang

**Affiliations:** Shaanxi Key Laboratory of Nanomaterials and Nanotechnology, Xi’an University of Architecture and Technology, Xi’an 710055, China; chenlp202203@xauat.edu.cn (L.C.);

**Keywords:** catalysts, metallic compounds, electron structure, lithium sulfur batteries, electrochemical performance

## Abstract

Although lithium–sulfur batteries possess the advantage of high theoretical specific capacity, the inevitable shuttle effect of lithium polysulfides is still a difficult problem restricting its application. The design of highly active catalysts to promote the redox reaction during charge–discharge and thus reduce the existence time of lithium polysulfides in the electrolyte is the mainstream solution at present. In particular, bimetallic compounds can provide more active sites and exhibit better catalytic properties than single-component metal compounds by regulating the electronic structure of the catalysts. In this work, bimetallic compounds-nitrogen-doped carbon nanotubes (NiCo)Se_2_-NCNT and (CuCo)Se_2_-NCNT are designed by introducing Ni and Cu into CoSe_2_, respectively. The (CuCo)Se_2_-NCNT delivers an optimized adsorption–catalytic conversion for lithium polysulfide, benefitting from adjusted electron structure with downshifted *d*-band center and increased electron fill number of Co in (CuCo)Se_2_ compared with that of (NiCo)Se_2_. This endows (CuCo)Se_2_ moderate adsorption strength for lithium polysulfides and better catalytic properties for their conversion. As a result, the lithium–sulfur batteries with (CuCo)Se_2_-NCNT achieve a high specific capacity of 1051.06 mAh g^−1^ at 1C and an enhanced rate property with a specific capacity of 838.27 mAh g^−1^ at 4C. The work provides meaningful insights into the design of bimetallic compounds as catalysts for lithium–sulfur batteries.

## 1. Introduction

Lithium–sulfur (Li-S) batteries have gotten a lot of attention since 2009 benefitting from their high theoretical specific capacity (1675 mAh g^−1^), energy density (2600 Wh kg^−1^), and application possibilities [1,2]. However, lithium polysulfide (LiPSs, Li_2_S*_x_*, 2 ≤ *x* ≤ 8) intermediates are generate during the discharge process, which is easily dissolved and suffers sluggish reaction kinetics. The accumulated LiPSs in the electrolyte will diffuse through the separator to the lithium anode and return to the cathode during the charging process, resulting in the so-called infamous shuttle effect [3]. This causes a loss of sulfur-active material and a decrease in capacity, slowing down the commercialization of batteries, which remains a major challenge [4]. In recent years, the solution strategies have focused on the design of catalysts for sulfur cathode or separator modification to adsorb the LiPSs and catalyze their conversion, reducing the shuttle effect and improving the charge–discharge property of Li-S batteries [5,6,7].

Currently, catalyst materials for Li-S batteries mainly include metal oxides, metal sulfides, metal nitrides, and metal phosphates with single metal components [8,9]. Considering the complex and multistep conversion of LiPSs in the process of charge–discharge and the difficulty of adjusting the electron structure of catalysts by single, it is extremely necessary to modify metal components for increasing active site and adjusting its catalytic activity with introduced metal ions [10,11,12]. The methods involve doping and the construction of bimetallic compounds [8,13,14,15]. Among them, doping modifications are deeply studied to boost the catalytic activity of catalysts for Li-S batteries, while bimetallic compounds should be further developed even though some reports. For example, NiCo_2_S_4_, NiCoP, Li_4_Ti_5_O_12_, Co_3_Mo_3_N, CoSn(OH)_6_, Ni_2_Co_4_P_3_, et al. had been designed, which delivered improved catalytic performance than corresponding to one component metallic compounds [16,17,18,19,20,21]. This could be ascribed to optimized chemical interaction between bimetallic compounds and LiPSs as well as catalytic activity, contributing from adjusted electron structure, such as *d* orbital electron filling and *d* band center [22,23,24,25,26]. This is because the LiPSs on catalysts include adsorption–conversion–desorption processes, weak adsorption cannot effectively trigger the reaction, while too strong adsorption passivates the active sites to hinder the subsequent reaction and moderate adsorption capacity will obtain the best catalytic performance [27]. Among them, *d* band center is an important parameter related to adsorption energy for LiPSs: the higher *d* band center, the stronger the chemical interaction, and vice versa [23]. For the *d* orbital fill number, the more it is filled, the weaker the binding capacity with LiPSs, and vice versa [28,29]. Introducing metal ions can optimize the adsorption–conversion–desorption process of LiPSs by adjusting the *d*-band center as well as electron filling of metal-based catalysts [21]. Ni element is widely selected for modifying Co-based metallic compounds due to their similar element characteristics, such as Ni-doped WS_2_, Ni-doped MoS_2_, and Ni_0.2_Mo_0.8_N [30,31,32]. Whether other elements can achieve better modification effects and the reason for this are worth exploring to promote the research of catalysts for Li-S batteries.

In this work, Ni and Cu are introduced into CoSe_2_ and construct bimetallic compounds-nitrogen-doped carbon nanotubes, (NiCo)Se_2_-NCNT and (CuCo)Se_2_-NCNT, respectively. Even though the (NiCo)Se_2_-NCNT presents a better adsorption effect, the (CuCo)Se_2_-NCNT delivers an improved catalytic performance compared to (NiCo)Se_2_-NCNT. Consequently, the (CuCo)Se_2_-NCNT modified separator (Figure S1) endows the corresponding Li-S batteries with a higher specific capacity and enhanced rate performance. The electron structure analysis reveals the better catalytic properties of (CuCo)Se_2_ resulting from a downshifted *d* band center and increased electron fill number.

## 2. Experimental

### 2.1. Preparation of Bimetallic Selenides

A total of 8 g of melamine and 0.1 g of anhydrous glucose were added into 60 mL of deionized water, after being stirred and mixed thoroughly. Ten mL of Ni(NO_3_)_2_·6H_2_O and Co(NO_3_)_2_·6H_2_O aqueous solution was then added drop by drop, in which the total mass of the two metal salt ions was 0.8 g and the molar ratio was 1:1. The mixture was then stirred at 60 °C until a dry powder was obtained. The powder was then heated at 800 °C for 3 h in N_2_ to obtain NiCo-NCNT. Finally, NiCo-NCNT was mixed with selenium powder at a mass ratio of 1:2 and heated at 400 °C for 2 h under nitrogen at a heating rate of 2 °C min^−1^ to obtain (NiCo)Se_2_-NCNT. (CuCo)Se_2_-NCNT was prepared by replacing Ni(NO_3_)_2_·6H_2_O with equimolar Cu(NO_3_)_2_·3H_2_O.

### 2.2. Preparation of Modified Separators and Sulfur Cathodes

(NiCo)Se_2_-NCNT/(CuCo)Se_2_-NCNT, acetylene black, and polyvinylidene fluoride (PVDF) were ground evenly at the mass ratio of 4:5:1, which was then stirred for 12 h with N-methylpyrrolidone (NMP). The prepared paste was coated on Celgard 2500 separator, and the modified separator was cut after vacuum drying at 60 °C for 12 h.

Sublimed sulfur powder and acetylene black were ground evenly at the mass ratio of 7:3 and heat treated at 155 °C under a nitrogen atmosphere for 12 h to obtain sulfur composite. Sulfur composite, acetylene black, and PVDF with a mass ratio of 7:2:1 were mixed in NMP and coated on carbon-coated aluminum foil. Sulfur cathode was obtained with a sulfur load of about 1.1 mg cm^−2^ after being dried at 60 °C for 12 h in a vacuum.

### 2.3. Characterization of Bimetallic Selenide Materials

X-ray diffraction (XRD) using Cu Kα radiation source, transmission electron microscopy (TEM), selected area electron diffraction (SAED), and energy dispersive spectroscopy were carried out to analyze the morphology, crystal structure, and composition of the prepared materials. X-ray photoelectron spectroscopy (XPS) was conducted to study the valence states of the materials. N_2_ adsorption/desorption analyzer was conducted to measure the pore size distribution.

### 2.4. Electrochemical Performance Test of Li-S Batteries

Sulfur cathode, modified separator, and lithium sheet were used to assemble 2032 coin batteries, using 1 M lithium bis(trifluoromethanesulfonyl) imide (LiTFSI) in DOL/DME with 2 wt% LiNO_3_ as electrolyte. Constant current charge–discharge tests were conducted on the NEWARE system at 1.7~2.8 V. Electrochemical impedance spectroscopy (EIS) was carried out at 10^2^~10^5^ Hz and ±5 mV. Cyclic voltammetry (CV) was performed on a CHI760E electrochemical workstation with a scanning speed of 0.1 mV s^−1^ at 1.7~2.8 V. Galvanostatic intermittent titration technique (GITT) was conducted to obtain open circuit voltage (OCV) with the current pulse of 30 min, and the quasi-open circuit voltage (QOCV) was obtained by standing for 1 h at 0.1C.

### 2.5. Analysis of Adsorption and Catalytic Properties of Bimetallic Selenides

#### 2.5.1. Adsorption Effect Evaluation

First, S and Li_2_S with a molar ratio of 5:1 were dissolved in 1,3-dioxolane (DOL) and dimethoxymethane (DME) (*v*:*v* = 1:1) to prepare Li_2_S_6_ solution. Then, an equal amount of bimetallic selenides was added to 2 mL Li_2_S_6_, respectively. The color change of the solution was recorded. And, after adsorption, the supernatant was removed and the sample was dried for the XPS test to analyze the change of valence state.

#### 2.5.2. CV test of Li_2_S_6_ Symmetric Batteries

The bimetallic selenides and PVDF were mixed at a mass ratio of 9:1 in NMP and coated on aluminum foil to make electrodes for symmetric batteries. A total of 15 μL of 0.5 M Li_2_S_6_ solution was used to assemble Li_2_S_6_ symmetric batteries. CV test was conducted in the range of −1.0~1.0 V at 10 mV s^−1^.

#### 2.5.3. Li_2_S Deposition and Decomposition Test

S and Li_2_S in a molar ratio of 7:1 were dissolved in tetraethylene glycol dimethyl ether at 60 °C to prepare 0.1 M Li_2_S_8_ solution. CR2032 coin batteries were assembled with the cathode sheets used for symmetric batteries, and lithium sheets as the anodes. The electrolyte on the cathode side was 20 μL of 0.1 M Li_2_S_8_, and that on the anode side was the same with Li-S batteries. The batteries were discharged at 0.113 mA to 2.06 V and then held at 2.05 V until the current was below 10^−2^ mA for Li_2_S deposition. The batteries were constantly discharged to 1.8 V, then potentiostatically discharged to a current lower than 0.01 mA, and then potentiostatically charged at 2.4 V for 10 h to evaluate Li_2_S decomposition ability.

## 3. Results and Discussion

### 3.1. Materials Characterization

**Figure 1:**
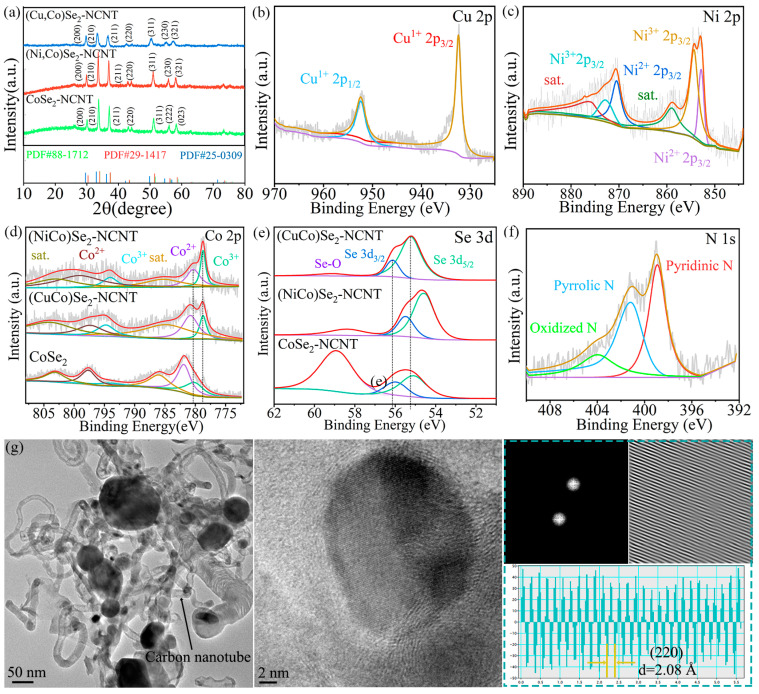
Physical and chemical property analyses of CoSe_2_-NCNT, (NiCo)Se_2_-NCNT, and (CuCo)Se_2_-NCNT: (**a**) XRD patterns, XPS: (**b**) Cu 2p of (CuCo)Se_2_-NCNT, (**c**) Ni 2p of (NiCo)Se_2_-NCNT, (**d**) comparison of Co 2p of three samples, (**e**) comparison of Se 3d, (**f**) N 1s of (CuCo)Se_2_-NCNT, (**g**) TEM, (**h**) HRTEM, (**i**)corresponding Fast Fourier transform patterns of (CuCo)Se_2_-NCNT.

XRD patterns of CoSe_2_-NCNT, (NiCo)Se_2_-NCNT, and (CuCo)Se_2_-NCNT are shown in Figure 1a, which correspond to PDF#88-1712, PDF#29-1417, and PDF#25-0309, respectively, proving the successful synthesis of bimetallic selenides. The three materials are cubic crystals with similar crystal structures, whose cell parameters are a = b = c = 5.859, a = b = c = 5.891, and a = b = c = 6.056, respectively. Moreover, the diffraction peaks of (NiCo)Se_2_-NCNT and (CuCo)Se_2_-NCNT are left-shifted compared to that of CoSe_2_-NCNT by 2° and 3°, respectively, indicating that the crystal plane spacing increases. To study the chemical state of different CoSe_2_-based catalysts, XPS tests were conducted. As shown in Figure 1b, the peaks of 932.1 and 952.1 eV are Cu^+^ 2p_3/2_ and Cu^+^ 2p_1/2_, and the peaks of 934.3 eV and 954.4 eV are Cu^2+^ 2p_3/2_ and Cu^2+^ 2p_1/2_ [33]. As for Ni 2p XPS spectrum of (NiCo)Se_2_-NCNT (Figure 1c), peaks at 854.40 eV and 856.00 eV are Ni^2+^ 2p_3/2_ and Ni^3+^ 2p_3/2_, peaks of 871.90 eV and 876.18 eV relate to Ni^2+^ 2p_1/2_ and Ni^3+^ 2p_1/2_ [34,35]. The effects of the introduction of Ni and Cu on the electronic structure is also compared, as displayed in Figure 1d,e. In comparison to CoSe_2_, the peaks of Co^2+^ 2p_1/2_ and Co^3+^ 2p_1/2_ of (NiCo)Se_2_-NCNT and (CuCo)Se_2_-NCNT are shifted to lower binding energies. The peaks of Se 3d of (CuCo)Se_2_-NCNT are upshifted, while that of (NiCo)Se_2_-NCNT is downshifted. This means the change of electron density CoSe_2_ resulting from the electron interaction of metal ions after the incorporation of Ni or Cu [19]. Figure 1f presents the N 1s spectrum, in which the Pyridinic N, Pyrrolic N, and Graphitic N are regarded as active sites to anchor LiPSs [36]. The microstructure and crystal structure of (CuCo)Se_2_-NCNT TEM are further studied by TEM. The material displays a one-dimensional tubular morphology with a diameter ranging from 10 to 60 nm, as shown in Figure 1g. In addition, most (CuCo)Se_2_ nanoparticles are located inside the carbon nanotubes. Moreover, the high-resolution TEM (HRTEM) (Figure 1h) shows that the nanoparticles are about 10–150 nm in diameter and wrapped in a carbon layer. The carbon nanotubes are in-situ generated during the high-temperature stage resulting from the catalytic action of metal particles with glucose as a carbon source. Furthermore, the nanoparticles are generally encapsulated in nanotubes and the diameters of the tubes are consistent with the nanoparticles. The size of nanoparticles has a great effect on the electrochemical performances, due to the fact that the smaller nanoparticles, the larger surface areas, meaning active sites to anchor LiPSs and catalyze their conversion [37]. The fast Fourier transform (FFT) corresponding to the nanoparticle in Figure 1h is presented in Figure 1i. The lattice spacing is 2.08 Å assigned to the (220) crystal plane of (CuCo)Se_2_. The SAED pattern (Figure S2) displays the diffraction ring of (002) of amorphous carbon and (220) lattice planes of (CuCo)Se_2_. According to the EDS result of (CuCo)Se_2_ (Table S1), the doping amount of Cu is 36.56 at.%. The pore structure of CoSe_2_-NCNT was characterized by N_2_ adsorption–desorption experiments. It can be seen from Figure S3 that the material mainly contains mesoporous of 4 nm.

### 3.2. Electrochemical Performance of Li-S Batteries

**Figure 2:**
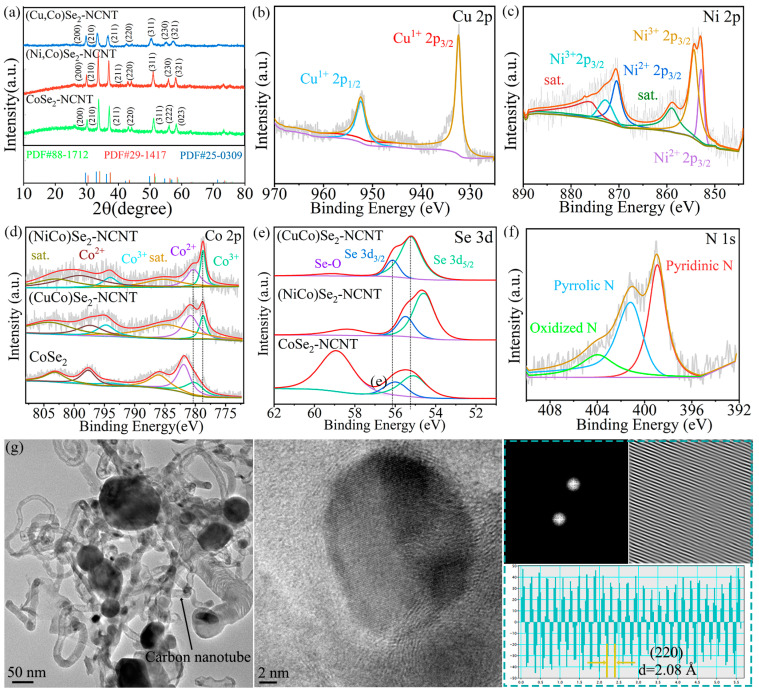
Electrochemical properties of various Li-S batteries: (**a**) cycling performance at 0.1C, (**b**) rate capability, (**c**) initial charge–discharge curves at 0.1C, charge–discharge curves of battery at different rate with (**d**) (CuCo)Se_2_-NCNT, (**e**) (NiCo)Se_2_-NCNT, (**f**) comparison of voltage difference (ΔE), (**g**) cycle performance at 1C, and (**h**) cycle stability of batteries with higher sulfur loading at 0.1C.

The cycle performance at 0.1C was first tested to compare the electrochemical performance of Li-S batteries with various separators (Figure 2a). The initial discharge capacities of Li-S batteries with (CuCo)Se_2_-NCNT, (NiCo)Se_2_-NCNT, and CoSe_2_-NCNT reach 1670.08, 1410.41, and 814.81 mAh g^−1^, respectively. After 140 cycles, the capacities are maintained at 634.74, 682.81, and 578.23 mAh g^−1^, respectively. In addition, the rate performance of the battery equipped with (CuCo)Se_2_-NCNT modified separator is much higher than that of the battery with (NiCo)Se_2_-NCNT and CoSe_2_-NCNT (Figure 2b). The discharge capacities at 0.1C, 0.2C, 0.5C, 1C, 2C, and 4C reach 1504.40, 1388.32, 1206.94, 1083.17, 959.77, and 838.27 mAh g^−1^, respectively, and the battery with (CuCo)Se_2_-NCNT achieves a discharge capacity of 1294.91 mAh g^−1^ when returns to 0.1C again, showing excellent rate performance. The initial charge and discharge curves at 0.1C are displayed in Figure 2c. Two platforms are presented during discharging, which correspond to the conversion of S_8_ into LiPSs and further reduction into Li_2_S, and their capacities are marked as Q_H_ and Q_L_, respectively. Li-S battery containing (CuCo)Se_2_-NCNT possess larger Q_H_ and Q_L_, meaning more S_8_ is converted to LiPSs and more Li_2_S are formed [38]. To compare the reaction kinetics of Li-S battery, the voltage difference is marked as ΔE, and the ΔE under different rates is compared, as presented in Figure 2d,e and Figure S4. Compared with Li-S batteries with (NiCo)Se_2_-NCNT and CoSe_2_-NCNT, that of (CuCo)Se_2_-NCNT exhibits the least polarization at various current densities (Figure 2f), meaning improved reaction kinetics [38]. The battery with (CuCo)Se_2_-NCNT achieves an initial capacity of 1051.06 mAh g^−1^ at 1C, which is much higher than that of (NiCo)Se_2_-NCNT (838.63 mAh g^−1^) and CoSe_2_-NCNT (657.44 mAh g^−1^). All in all, the Li-S batteries with bimetallic compounds exhibit superior electrochemical performance, including higher specific capacity, more stable cycle performance, and optimal reaction kinetics, especially (CuCo)Se_2_, as displayed in Table S2. This means that the (CuCo)Se_2_ is more conducive to the application of Li-S batteries for higher capacity and longer cycle life.

### 3.3. Comparison of Adsorption–Catalytic Properties of Bimetallic Selenides

**Figure 3:**
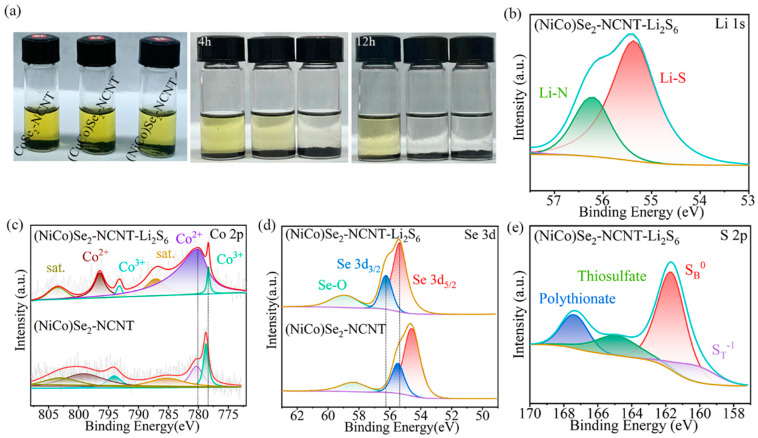
(**a**) Comparison of visible adsorption effect; XPS spectra of (NiCo)Se_2_-NCNT after adsorption test: (**b**) Li 1s, (**c**) Co 2p, (**d**) Se 3d, (**e**) S 2p.

To compare the adsorption capacity of the three materials for LiPSs, equal amounts of (NiCo)Se_2_-NCNT, (CuCo)Se_2_-NCNT, and CoSe_2_-NCNTy were added to Li_2_S_6_ solution, and the color change was recorded. As shown in Figure 3a, the solution containing (NiCo)Se_2_-NCNT began to fade first, suggesting the best adsorption effect, followed by (CuCo)Se_2_-NCNT. The (NiCo)Se_2_-NCNT after adsorption was analyzed by XPS. Figure 3b shows that a Li-N bond is formed between the catalyst and Li_2_S_6_ besides the Li-S bond of Li_2_S_6_. Furthermore, as displayed in Figure 3c,d, after adsorbing Li_2_S_6_, the peak of Co 2p moves towards lower binding energy, while the peak of Se 3d moves in the direction of higher binding energy compared to the pristine sample, indicating the occurred chemical interaction of (NiCo)Se_2_-NCNT and LiPSs as the electron transfer between them [39]. In addition, the S 2p XPS (Figure 3e) displays the existence of polythionate and thiosulfate, suggesting the oxidation of polysulfides, which is conductive to anchoring LiPSs.

**Figure 4:**
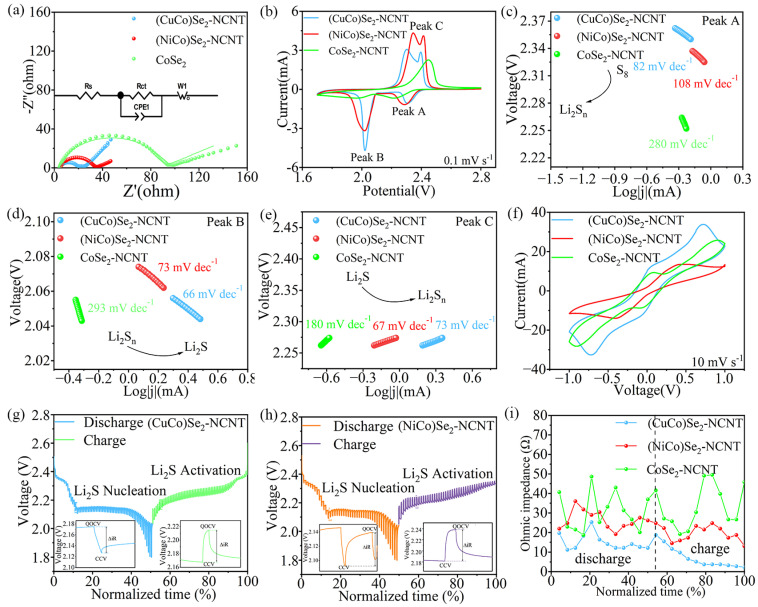
Catalytic performance analyses: (**a**) EIS, (**b**) CV at 0.1 mV s^−1^, Tafel curves of (**c**) Peak A, (**d**) Peak B, (**e**) Peak C, (**f**) CV of Li_2_S_6_ symmetrical cell at scanning rate of 10 mV s^−1^, GITT plots of Li-S batteries with (**g**) (CuCo)Se_2_-NCNT, (**h**) (NiCo)Se_2_-NCNT, and (**i**) comparison of impedance during charging–discharging.

The EIS spectra of different Li-S batteries present semicircular in high-frequency regions and oblique in low-frequency regions (Figure 4a). The value of the semicircle radius depends on the resistance generated by the charge transfer between the electrolyte and the cathode, and the slope of the diagonal depends on the rate of ion diffusion [35]. It can be seen that the battery of (CuCo)Se_2_-NCNT delivers the smallest resistance and the largest Li^+^ diffusion rate in comparison to the other samples. Furthermore, the reduction peaks (Peak A) around 2.3 V and 2.0 V (Peak B) in CV curves are attributed to S_8_ reduced to Li_2_S*_x_* (4 ≤ *x* ≤ 8) and then Li_2_S/Li_2_S_2_ (Figure 4b) [5]. The oxidation peak (Peak C) is related to the conversion from Li_2_S to LiPSs/S_8_ [40]. (CuCo)Se_2_-NCNT endows the corresponding battery with the optimized potential difference and highest peak current compared with CoSe_2_-NCNT and (NiCo)Se_2_-NCNT, meaning an improvement in sulfur utilization and reaction kinetics [41]. Tafel slopes were further analyzed according to CV curves to assess the catalytic capacity (Figure 4c–e). The Tafel slopes of the battery with (CuCo)Se_2_-NCNT are 82 and 66 mV dec^−1^ for Peak A and Peak B, and 73 mV dec^−1^ for Peak C. It is much smaller than (NiCo)Se_2_-NCNT (108, 73, 67 mV dec^−1^) and CoSe_2_-NCNT (280, 293, 180 mV dec^−1^). This means that (CuCo)Se_2_-NCNT can effectively promote the conversion of sulfur species [42]. As for the CV of Li_2_S_6_ symmetric batteries (Figure 4f), the peaks are related to the redox of Li_2_S_6_. The battery with (CuCo)Se_2_-NCNT delivers the largest peak current, indicating better catalytic activity than other samples, thus enhancing LiPSs conversion kinetics [19,43]. GITT was also applied to analyze the ohmic impedance during charge–discharge, and the calculation basises of ohmic impedance is shown in Figure 4g,h and Figure S5, and Equation (S1) [44]. It can be seen from Figure 4i that the battery with (CuCo)Se_2_-NCNT presents the lowest impedance in comparison to (NiCo)Se_2_-NCNT and CoSe_2_-NCNT, meaning facilitated reaction kinetics [45].

**Figure 5:**
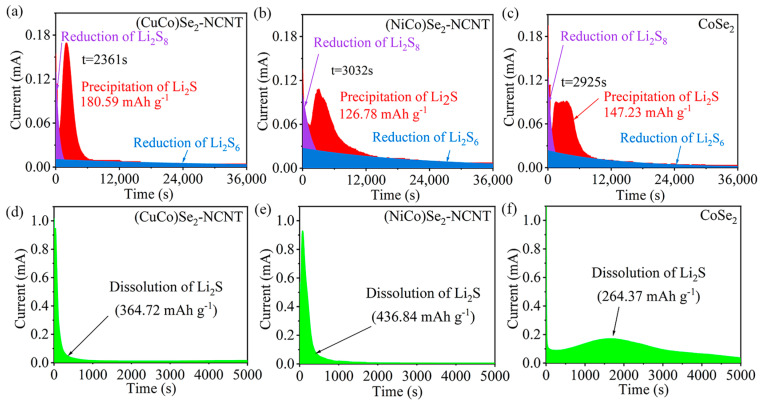
Potentiostatic discharging curves of Li_2_S_8_ solution on different catalysts at 2.05 V: (**a**) (CuCo)Se_2_-NCNT; (**b**) (NiCo)Se_2_-NCNT; (**c**) CoSe_2_-NCNT, potentiostatic charging curves of Li_2_S_8_ solution at 2.4 V on different catalysts: (**d**) (CuCo)Se_2_-NCNT and (**e**) (NiCo)Se_2_-NCNT, (**f**) CoSe_2_-NCNT.

To analyze the catalytic effect of various bimetallic selenides on the liquid–solid conversion from LiPSs to Li_2_S, nucleation experiments of Li_2_S were carried out at 2.05 V. The (CuCo)Se_2_-NCNT achieves a larger Li_2_S deposition capacity of 180.59 mAh g^−1^ than (NiCo)Se_2_-NCNT (126.78 mAh g^−1^) and CoSe_2_-NCNT (147.23 mAh g^−1^) (Figure 5a–c). In the meanwhile, Li_2_S has a faster nucleation response (2361 s) on (CuCo)Se_2_-NCNT than on (NiCo)Se_2_-NCNT and CoSe_2_-NCNT. This suggests that (CuCo)Se_2_-NCNT can significantly promote the nucleation and growth of Li_2_S [21]. Similarly, the Li_2_S decomposition experiments were carried out at 2.4 V to evaluate the decomposition ability of Li_2_S under different catalysts. The Li_2_S decomposition capacity on the surface of (NiCo)Se_2_-NCNT (436.84 mAh g^−1^) is superior to (CuCo)Se_2_-NCNT (364.72.8 mAh g^−1^) and CoSe_2_-NCNT (264.37 mAh g^−1^) (Figure 5d–f), presenting a good catalytic effect on the decomposition of Li_2_S [46].

**Figure 6:**
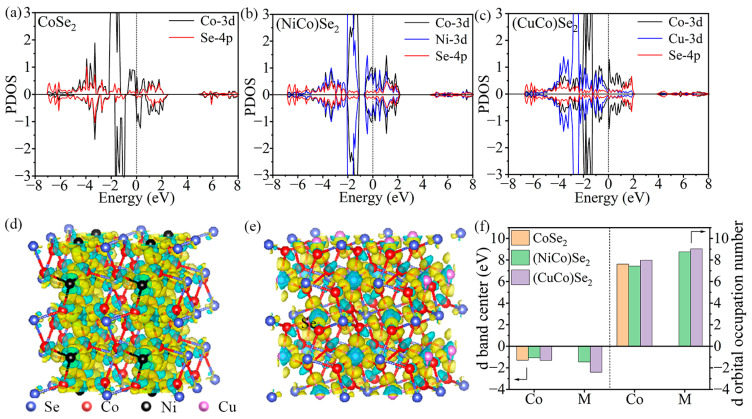
Theoretical analyses of *d* orbital electronic structure. DOS of: (**a**) CoSe_2_, (**b**) (NiCo)Se_2_, (**c**) (CuCo)Se_2_, Electron density difference of (**d**) (NiCo)Se_2_, (**e**) (CuCo)Se_2_, (**f**) Comparison of *d* orbital electronic structure parameters.

To reveal the fundamental reason for the superior catalytic performance of (CuCo)Se_2_, the electronic structure was analyzed by first-principles calculation. The theoretical calculation structure models of the three samples and the model structure parameters are displayed in Figure S6 and Table S3. (CuCo)Se_2_ presents larger lattice parameters than the other two, which is in line with the XRD analysis result. Moreover, the density of states of different materials was also calculated. The (CuCo)Se_2_ presents a higher density of states around the Fermi level in comparison to CoSe_2_ and (NiCo)Se_2_ (Figure 6a), meaning a better electron conductivity, which can help to accelerate the electrochemical reaction [8,47,48]. Charge density difference analyses are further displayed in Figure 6b,c, the yellow region stands for charge accumulation and the cyan region means charge depletion. As a result, electron consumption occurs around Ni and Cu, suggesting charge transfer from Ni/Cu to other ions and causing charge redistribution. Moreover, the *d* orbital electronic structure of Co and incorporated metal ions is presented in Figure 6d. Compared to CoSe_2_, the *d* band center of Co in (NiCo)Se_2_ is upshifted, while that of Co in (CuCo)Se_2_ is downshifted. Meanwhile, the *d* band center of Ni is also higher than that of Cu. According to *d* band center theory, the upshifted *d* band center, the higher the adsorption energy [28,49]. However, the catalyzing LiPSs conversion involves the adsorption–catalysis–desorption process, and too strong adsorption could inhibit the desorption process and occupy active sites, which is not conductive to further reaction [23,27,50]. In addition, Cu increases the *d* orbital occupation number of Co while the Ni decreases it (Figure 6d and Table S4), thus improving the desorption and catalytic property of (CuCo)Se_2_ for LiPSs [21,26]. Consequently, the introduction of Cu into CoSe_2_ endows the (CuCo)Se_2_ with a relatively low *d* band center and *d* orbital occupation number of Co, which can render it to achieve proper adsorption energy for LiPSs and catalyze their conversion, promoting the electrochemical properties of Li-S batteries.

In addition to being used in Li-S batteries, bimetallic selenides can also be used in other types of batteries (lithium-ion batteries, sodium-ion batteries, magnesium-ion batteries, et al.), various supercapacitors, electrocatalysts for hydrogen evolution reaction, oxygen evolution reaction, etc. [51,52,53,54,55,56,57]. Therefore, the designed bimetallic selenides in this paper can also be extended to other fields.

## 4. Conclusions

In summary, Ni and Cu were successfully introduced to CoSe_2_ and constructting bimetallic compounds (NiCo)Se_2_ and (CuCo)Se_2_. The electron structure of (NiCo)Se_2_ and (CuCo)Se_2_ were adjusted to optimize the adsorption–conversion of LiPSs. To be specific, the Ni rendered the *d* band center of Co in (NiCo)Se_2_ upshifted, while Cu downshifted it. In the meanwhile, *d* orbital occupation number of Co in (CuCo)Se_2_ was increased but that of (NiCo)Se_2_ was decreased. The downshifted *d* band center and increase of *d* orbital occupation number of Co in (CuCo)Se_2_ could endow the LiPSs with moderate adsorption energy, optimizing their adsorption–conversion process. In contrast, too strong adsorption of (NiCo)Se_2_ would inhibit the desorption of LiPSs and was not conductive to further catalyze the LiPSs conversion. Consequently, the (CuCo)Se_2_ delivered better catalytic properties for LiPSs and endowed the corresponding Li-S batteries with improved electrochemical performance.

## Data Availability

The data that support the findings of this study are available on request from the corresponding author.

## Supplementary Materials

The following supporting information can be downloaded at: https://www.mdpi.com/article/10.3390/ma17133075/s1, Figure S1. Scheme of Li-S battery with modified separator. Figure S2. Selected area electron diffraction (SAED) pattern of (CuCo)Se_2_. Figure S3. Pore size distribution of CoSe_2_-NCNT. Figure S4. Charge-discharge curves of battery with CoSe_2_-NCNT at different rate. Figure S5. GITT plots of Li-S batteries with CoSe_2_-NCNT. Figure S6. Theoretical calculation structure model of (a) CoSe_2_, (b) (NiCo)Se_2_, (c) (CuCo)Se_2_. Table S1. The content of each element in (CuCo)Se_2_. Table S2. Comparison of electrochemical performance of Li-S batteries with different catalysts. Table S3. Theoretical model structural parameters of different materials. Table S4. *d* orbital electronic structure parameters of different (MCo)Se_2_.

## References

[1] Liang Z., Shen J., Xu X., Li F., Liu J., Yuan B., Yu Y., Zhu M. (2022). Advances in the development of single-atom catalysts for high-energy-density lithium-sulfur batteries. Adv. Mater..

[2] Liu T., Hu H., Ding X., Yuan H., Jin C., Nai J., Liu Y., Wang Y., Wan Y., Tao X. (2020). 12 years roadmap of the sulfur cathode for lithium sulfur batteries. Energy Storage Mater..

[3] Long J.J., Yu H., Liu W.B. (2024). Structure engineering of cathode host materials for Li-S batteries. Rare Metals.

[4] Lv Z.C., Wang P.F., Wang J.C., Tian S.H., Yi T.F. (2023). Key challenges, recent advances and future perspectives of rechargeable lithium-sulfur batteries. J. Ind. Eng. Chem..

[5] Cao G., Duan R., Li X. (2023). Controllable catalysis behavior for high performance lithium sulfur batteries: From kinetics to strategies. EnergyChem.

[6] Zhou L., Danilov D.L., Eichel R.A., Notten P.H.L. (2021). Host materials anchoring polysulfides in Li-S batteries reviewed. Adv. Energy Mater..

[7] Han J., Wang P., Zhang H., Song N., An X., Xi B., Xiong S. (2024). Performance optimization of chalcogenide catalytic materials in lithium-sulfur batteries: Structural and electronic engineering. Chin. Chem. Lett..

[8] Chen L., Cao G., Li Y., Zu G., Duan R., Bai Y., Xue K., Fu Y., Xu Y., Wang J. (2024). review on engineering transition metal compound catalysts to accelerate the redox kinetics of sulfur cathodes for lithium-sulfur batteries. Nano-Micro Lett..

[9] Wang J., Du R., Yu C., Xu C., Shi Z. (2022). Application of transition metal compounds in cathode materials for lithium-sulfur battery. Ionics.

[10] Shi F., Yu J., Chen C., Lau S.P., Lv W., Xu Z.-L. (2022). Advances in understanding and regulation of sulfur conversion processes in metal-sulfur batteries. J. Mater. Chem. A.

[11] Du S., Yu Y., Liu X., Lu D., Yue X., Liu T., Yin Y., Wu Z. (2024). Catalytic engineering for polysulfide conversion in high-performance lithium-sulfur batteries. J. Mater. Sci. Technol..

[12] Yao Y., Chen J., Niu R., Zhao Z., Wang X. (2023). High-entropy materials: Features for lithium-sulfur battery applications. Metals.

[13] Jiang Y.C., Arshad H.M.U., Li H.J., Liu S., Li G.R., Gao X.P. (2021). Crystalline multi-metallic compounds as host materials in cathode for lithium-sulfur batteries. Small.

[14] Huang S., Wang Z., Von Lim Y., Wang Y., Li Y., Zhang D., Yang H.Y. (2021). Recent advances in heterostructure engineering for lithium-sulfur batteries. Adv. Energy Mater..

[15] Wang J., Han W.-Q. (2022). A review of heteroatom doped materials for advanced lithium-sulfur batteries. Adv. Funct. Mater..

[16] Li S., Xu P., Aslam M.K., Chen C., Rashid A., Wang G., Zhang L., Mao B. (2020). Propelling polysulfide conversion for high-loading lithium-sulfur batteries through highly sulfiphilic NiCo_2_S_4_ nanotubes. Energy Storage Mater..

[17] Duan J., Zou Y., Li Z., Long B., Du Y. (2019). Hollow quasi-polyhedron structure of NiCoP with strong constraint sulfur effect for lithium sulfur battery. Electroanal. Chem..

[18] Zhao S., Li Y., Zhang F., Guo J. (2019). Li_4_Ti_5_O_12_ nanowire array as a sulfur host for high performance lithium sulfur battery. J. Alloys Compd..

[19] Yan Y., Li H., Cheng C., Yan T., Gao W., Mao J., Dai K., Zhang L. (2021). Boosting polysulfide redox conversion of Li-S batteries by one-step-synthesized Co-Mo bimetallic nitride. J. Energy Chem..

[20] Wang X., Han J., Luo C., Zhang B., Ma J., Li Z., He Y.-B., Yang Q.-H., Kang F., Lv W. (2021). Coordinated adsorption and catalytic conversion of polysulfides enabled by perovskite bimetallic hydroxide nanocages for lithium-sulfur batteries. Small.

[21] Shen Z., Cao M., Zhang Z., Pu J., Zhong C., Li J., Ma H., Li F., Zhu J., Pan F. (2020). Efficient Ni_2_Co_4_P_3_ nanowires catalysts enhance ultrahigh-loading lithium-sulfur conversion in a microreactor-like battery. Adv. Funct. Mater..

[22] Li Y., Zhang J., Chen Q., Xia X., Chen M. (2021). Emerging of heterostructure materials in energy storage: A review. Adv. Mater..

[23] Wang B., Wang L., Ding D., Zhai Y., Wang F., Jing Z., Yang X., Kong Y., Qian Y., Xu L. (2022). Zinc-assisted cobalt ditelluride polyhedra inducing lattice strain to endow efficient adsorption-catalysis for high-energy lithium-sulfur batteries. Adv. Mater..

[24] Shang C., Li G., Wei B., Wang J., Gao R., Tian Y., Chen Q., Zhang Y., Shui L., Zhou G. (2021). Dissolving vanadium into titanium nitride lattice framework for rational polysulfide regulation in Li-S batteries. Adv. Energy Mater..

[25] Wang L., Hu Z., Wan X., Hua W., Li H., Yang Q.H., Wang W. (2022). Li_2_S_4_ anchoring governs the catalytic sulfur reduction on defective SmMn_2_O_5_ in lithium-sulfur battery. Adv. Energy Mater..

[26] Cheng Z., Wang Y., Zhang W., Xu M. (2020). Boosting polysulfide conversion in lithium-sulfur batteries by cobalt-doped vanadium nitride microflowers. ACS Appl. Energy Mater..

[27] Shen Z., Jin X., Tian J., Li M., Yuan Y., Zhang S., Fang S., Fan X., Xu W., Lu H. (2022). Cation-doped ZnS catalysts for polysulfide conversion in lithium-sulfur batteries. Nat. Catal..

[28] Wang M., Fan L., Sun X., Guan B., Jiang B., Wu X., Tian D., Sun K., Qiu Y., Yin X. (2020). Nitrogen-doped CoSe_2_ as a bifunctional catalyst for high areal capacity and lean electrolyte of Li-S battery. ACS Energy Lett..

[29] Sun D., Zhou J., Rao D., Zhu L., Niu S., Cai J., Fang Y., Liu Y., Liu X., Zang Y. (2021). Regulating the electron filling state of d orbitals in Ta-based compounds for tunable lithium-sulfur chemistry. Sustain. Mater. Techno..

[30] Al-Tahan M.A., Dong Y., Shrshr A.E., Liu X., Zhang R., Guan H., Zhang J. (2022). Enormous-sulfur-content cathode and excellent electrochemical performance of Li-S battery accouched by surface engineering of Ni-doped WS_2_@rGO nanohybrid as a modified separator. J. Colloid Interf. Sci..

[31] Zhang R., Dong Y., Al-Tahan M.A., Zhang Y., Wei R., Ma Y., Yang C., Zhang J. (2021). Insights into the sandwich-like ultrathin Ni-doped MoS_2_/rGO hybrid as effective sulfur hosts with excellent adsorption and electrocatalysis effects for lithium-sulfur batteries. J. Energy Chem..

[32] Zhang H., Dai R., Zhu S., Zhou L., Xu Q., Min Y. (2022). Bimetallic nitride modified separator constructs internal electric field for high-performance lithium-sulfur battery. Chem. Eng. J..

[33] Jiang Z., Yuan Y., Tan L., Li M., Peng K. (2023). Self-reconstruction of (CoNiFeCuCr)Se high-entropy selenide for efficient oxygen evolution reaction. Appl. Surf. Sci..

[34] Tian L., Zhang Z., Liu S., Li G., Gao X. (2022). High-entropy spinel oxide nanofibers as catalytic sulfur hosts promise the high gravimetric and volumetric capacities for lithium-sulfur batteries. Energy Environ. Mater..

[35] Yao W., Tian C., Yang C., Xu J., Meng Y., Manke I., Chen N., Wu Z., Zhan L., Wang Y. (2022). P-doped NiTe_2_ with Te-vacancies in lithium-sulfur batteries prevents shuttling and promotes polysulfide conversion. Adv. Mater..

[36] Ao J., Xie Y., Lai Y., Yang M., Xu J., Wu F., Cheng S., Wang X. (2023). CoSe_2_ nanoparticles-decorated carbon nanofibers as a hierarchical self-supported sulfur host for high-energy lithium-sulfur batteries. Sci. China Mater..

[37] Zhang X., Zhang Y., Wei X., Wei C., Song Y. (2021). A review of size engineering-enabled electrocatalysts for Li-S chemistry. Nanoscale Adv..

[38] Gu Z., Cheng C., Yan T., Liu G., Jiang J., Mao J., Dai K., Li J., Wu J., Zhang L. (2021). Synergistic effect of Co_3_Fe_7_ alloy and N-doped hollow carbon spheres with high activity and stability for high-performance lithium-sulfur batteries. Nano Energy.

[39] Jiang B., Qiu Y., Tian D., Zhang Y., Song X., Zhao C., Wang M., Sun X., Huang H., Zhao C. (2021). Crystal facet engineering induced active tin dioxide nanocatalysts for highly stable lithium-sulfur batteries. Adv. Energy Mater..

[40] Yao W., Zheng W., Xu J., Tian C., Han K., Sun W., Xiao S. (2021). ZnS-SnS@NC heterostructure as robust lithiophilicity and sulfiphilicity mediator toward high-rate and long-life lithium-sulfur batteries. ACS Nano..

[41] Zhang Y., Qiu Y., Fan L., Sun X., Jiang B., Wang M., Wu X., Tian D., Song X., Yin X. (2023). Dual-atoms iron sites boost the kinetics of reversible conversion of polysulfide for high-performance lithium-sulfur batteries. Energy Storage Mater..

[42] Song X., Tian D., Qiu Y., Sun X., Jiang B., Zhao C., Zhang Y., Fan L., Zhang N. (2022). Accelerating sulfur redox reactions by topological insulator Bi_2_Te_3_ for high-performance Li-S batteries. Adv. Funct. Mater..

[43] Cai D., Liu B., Zhu D., Chen D., Lu M., Cao J., Han W. (2020). Ultrafine Co_3_Se_4_ nanoparticles in nitrogen-doped 3D carbon matrix for high-stable and long-cycle-life lithium sulfur batteries. Adv. Energy Mater..

[44] Wen K., Huang L., Qu L., Deng T., Men X., Chen L., Wang J. (2023). g-C_3_N_4_/MoO_3_ composite with optimized crystal face: A synergistic adsorption-catalysis for boosting cathode performance of lithium-sulfur batteries. J. Colloid Interf. Sci..

[45] Qu L., Wang J., Chen L., Men X., Deng T., Wen K., Huang L. (2023). Accelerated polysulfide conversion using nitrogen-doped carbon nanofibers embedded with V_2_O_3_ as interlayers for lithium-sulfur batteries. Ionics.

[46] Guo Y., Li J., Yuan G., Guo J., Zheng Y., Huang Y., Shao H. (2023). Elucidating the volcanic-type catalytic behavior in lithium-sulfur batteries via defect engineering. ACS Nano..

[47] Song X., Qu Y., Zhao L., Zhao M. (2021). Monolayer Fe_3_GeX_2_ (X = S, Se, and Te) as highly efficient electrocatalysts for lithium-sulfur batteries. ACS Appl. Mater. Interfaces.

[48] Shi L., Fang H., Yang X., Xue J., Li C., Hou S., Hu C. (2021). Fe-cation doping in NiSe_2_ as an effective method of electronic structure modulation towards high-performance lithium-sulfur batteries. ChemSusChem.

[49] Xu H., Hu R., Zhang Y., Yan H., Zhu Q., Shang J., Yang S., Li B. (2021). Nano high-entropy alloy with strong affinity driving fast polysulfide conversion towards stable lithium sulfur batteries. Energy Storage Mater..

[50] Zhang W., Pan H., Han N., Feng S., Zhang X., Guo W., Tan P., Xie S., Zhou Z., Ma Q. (2023). Balancing adsorption, catalysis, and desorption in cathode catalyst for Li-S batteries. Adv. Energy Mater..

[51] Seo H.Y., Choi J.H., Kim Y.B., Cho J.S., Kang Y.C., Park G.D. (2023). Tailoring the shell thickness of yolk-shell structured carbon microspheres: Applications in metal selenide and carbon composite microspheres for enhanced sodium ion storage properties. J. Mater. Chem. A.

[52] Wang Z., Zhang F., Chen S., Gao S., Wang L., Liu X., Li M., Shangguan E. (2024). Facile preparation of bimetallic cobalt-copper selenide nanosheets as stable cathode materials for rechargeable magnesium batteries. J. Alloys Compd..

[53] Meshesha M.M., Chanda D., Jang S.G., Yang B.L. (2023). Enhancing cobalt-based bimetallic selenide performance for urea and water electrolysis through interface engineering. Chem. Eng. J..

[54] Chae S.H., Muthurasu A., Kim T., Kim J.S., Khil M.S., Lee M., Kim H., Lee J.Y., Kim H.Y. (2021). Templated fabrication of perfectly aligned metal-organic framework-supported iron-doped copper-cobalt selenide nanostructure on hollow carbon nanofibers for an efficient trifunctional electrode material. Appl. Catal. B Environ..

[55] Lee S.J., Lee S.H., Ha J.S., Kim I.T., Park S.H., Park H.K., Park H.J., Kang B.K., Park Y.S. (2024). Highly Active cobalt-copper-selenide electrocatalysts for solar-driven oxygen evolution reaction: An electrochemical activation energy aspect. ACS Sustain. Chem. Eng..

[56] Hui X., Zhao J., Mao J., Zhao H. (2023). Reduced graphene oxide-wrapped copper cobalt selenide composites as anode materials for high-performance lithium-ion batteries. Colloids Surf. A Physicochem. Eng. Asp..

[57] Sun P., Zhang J., Huang J., Wang L., Wang P., Cai C., Lu M., Yao Z., Yang Y. (2021). Bimetallic MOF-derived (CuCo)Se nanoparticles embedded in nitrogen-doped carbon framework with boosted electrochemical performance for hybrid supercapacitor. Mater. Res. Bull..

