# Supplementary material for: Optimized Adsorption–Catalytic Conversion for Lithium Polysulfides by Constructing Bimetallic Compounds for Lithium–Sulfur Batteries
Source: Materials (Basel). 2024 Jun 22;17(13):3075. doi: 10.3390/ma17133075 (PMC11242168; doi:10.3390/ma17133075)
Supplement: Supplementary file 1 [file materials-17-03075-s001.zip › materials-3039826-supplementary.pdf]

Supporting Information

Optimized *d* orbital electronic structure by constructing bimetallic compounds for catalytic effect of lithium-sulfur batteries

Liping Chen, Runhua Wang, Nan Li, Yang Bai, Yimo Zhou, Yonghong Fu, Juan Wang*

Shaanxi Key Laboratory of Nanomaterials and Nanotechnology, Xi'an Key Laboratory of Clean Energy, Xi'an University of Architecture and Technology, Xi'an 710055, P.R. China

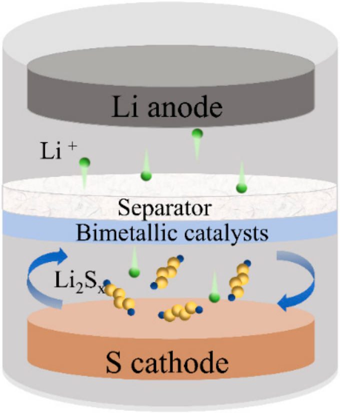

FigureS1. Scheme of Li-S battery with modified separator

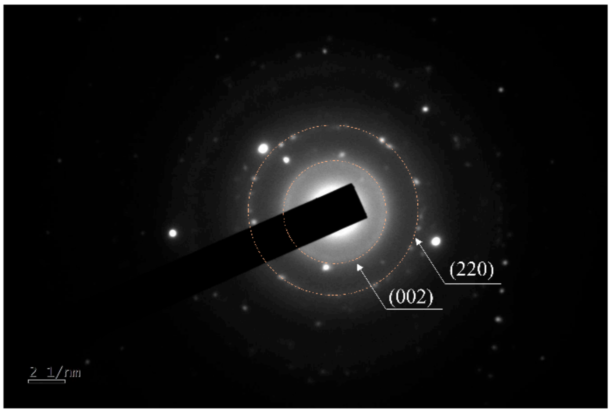

Figure S2. Selected area electron diffraction (SAED) pattern of $(Cu,Co)Se_2$

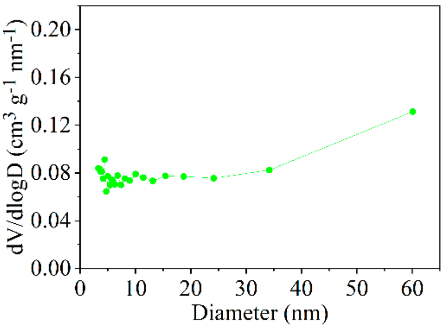

FigureS3. Pore size distribution of $CoSe_2$-NCNT

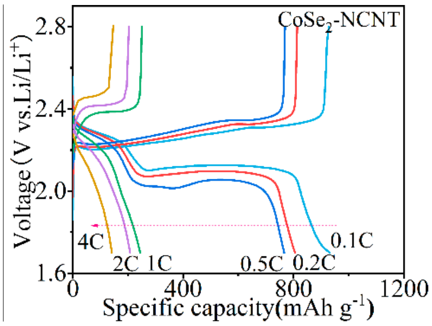

FigureS4. Charge-discharge curves of battery with $CoSe_2$-NCNT at different rate

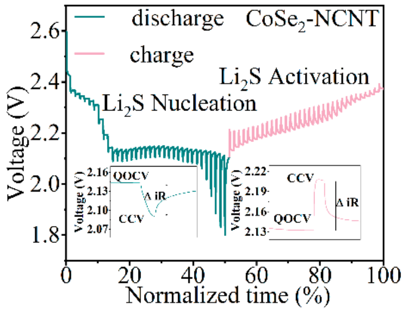

FigureS5. GITT plots of Li-S batteries with $CoSe_2$-NCNT

$$\Delta R_{\mathrm{intern}}\,(\Omega) = |\Delta V_{QOCV\text{-}CCV}| \,/\, I_{applied} \qquad \mathrm{S(1)}$$

where $\Delta R_{\mathrm{intern}}$ (Ω) refers to the internal resistances of Li-S batteries, QOCV and CCV are the quasi-open-circuit voltage and closed-circuit-voltage, respectively. And the $\Delta V_{QOCV\text{-}CCV}$ represents their difference, $I_{applied}$ stands for the applied current.

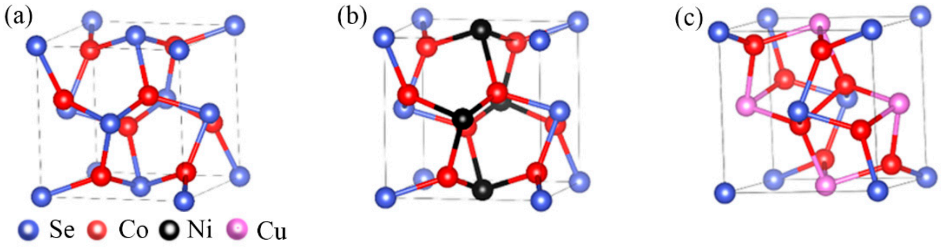

FigureS6. Theoretical calculation structure model of (a) $CoSe_2$, (b) $(Ni,Co)Se_2$, (c) $(Cu,Co)Se_2$

Table S1 The content of each element in $(CuCo)Se_2$

Elements	wt.%	at.%
C	35.88	73.83
N	3.15	5.55
Co	10.02	4.20
Cu	6.23	2.42
Se	44.72	14.00

Table S2 Comparison of electrochemical performance of Li-S batteries with different catalysts

Samples	Specific capacity (mAh g^{-1})			Capacity decay rate	ΔE (mV)	
	0.1C	4C	1C	1C	2C	4C
$CoSe_2$	814.81	142.18	657.44	0.084%	580	730
$(NiCo)Se_2$	1410.41	395.68	838.63	0.123%	630	690
$(CuCo)Se_2$	1670.08	838.27	1051.06	0.086%	360	600

Table S3 Theoretical model structural parameters of different materials

	Lattice A	Lattice B	Lattice C
$CoSe_2$	5.848	5.848	5.848
$(Ni,Co)Se_2$	5.900	5.886	5.883
$(Cu,Co)Se_2$	6.043	5.954	5.954

Table S4 *d* orbital electronic structure parameters of different $(MCo)Se_2$

Materials	*d* band center（eV）		*d* orbital occupation number	
	Co	M	Co	M
$CoSe_2$	-1.292	-	7.61	-
$(NiCo)Se_2$	-1.070	-1.461	7.43	8.75
$(CuCo)Se_2$	-1.314	-2.403	7.98	9.03
